# AddaVax Formulated with PolyI:C as a Potential Adjuvant of MDCK-based Influenza Vaccine Enhances Local, Cellular, and Antibody Protective Immune Response in Mice

**DOI:** 10.1208/s12249-021-02145-0

**Published:** 2021-11-11

**Authors:** Xuanxuan Nian, Jiayou Zhang, Tao Deng, Jing Liu, Zheng Gong, Chuanshuo Lv, Luyao Yao, Junying Li, Shihe Huang, Xiaoming Yang

**Affiliations:** 1 National Engineering Technology Research Center for Combined Vaccines, Wuhan, 430207 China; 2 Wuhan Institute of Biological Products Co. Ltd., Wuhan, 430207 China; 3 China National Biotec Group Company Limited, No.4, Huixin East Street, Chaoyang District , Beijing, 100029 China

**Keywords:** influenza, adjuvant, cellular response, humoral response

## Abstract

Poor immune responses to inactivated influenza vaccine can be improved by effective and safe adjuvants to increase antibody titers and cellular protective response. In our study, AddaVax and PolyI:C combined adjuvant (AP adjuvant) were used for influenza vaccine development. After immunizing BALB/c mice and Wistar rats intramuscularly, Split inactivated H3N2 vaccine adjuvanted with AP elicited higher serum hemagglutination-inhibition antibodies and IgG titers. We demonstrated that AP induced a transient innate immune cytokines production at the injection site, induced H3N2 uptake by DCs, increased recruitment of monocytes and DCs in LNs, and promoted H3N2 vaccine migration; AP facilitated vaccines to induce a vigorous adaptive immune response. Besides, AP showed good safety as shown by lymph nodes (LNs) size, spleens index of BALB/c mice, and weight changes and C-reaction protein level of BALB/c mice and Wistar rats after repeated administration of high-dose vaccine with or without adjuvant. These findings indicate that AP is a potential novel adjuvant and can be used as a safe and effective adjuvant for MDCK-based influenza inactivated vaccine to induce cellular and antibody protective response.

## INTRODUCTION

Seasonal influenza is a global public health threat, and vaccination is the most effective and dependable method for managing influenza epidemics (1). Madin-Darby canine kidney (MDCK) cells are susceptible to influenza virus infection and support virus replication; thus, they are used for cell-based influenza vaccines production (2). However, most inactivated or subunit influenza vaccines are poorly immunogenic and are ineffective at producing high levels of vaccine-specific serum antibodies in humans. Adjuvants and delivery systems used to improve immunogenicity of vaccines must be safe and effective (3). Several adjuvants for influenza vaccine have been used in humans, and others are in the early stages of clinical studies. In addition, many adjuvants in the preclinical research stage present promising results(4).

AddaVax, a squalene-based oil-in-water (w/o) nano-emulsion, similar to MF59, has been approved as a seasonal influenza vaccine component in Europe for people aged 65 and older(5). AddaVax is used in experimental vaccines to enhance antibody titers (6–8). Toll-like receptors (TLRs), key pathogen sensors that modulate the host’s innate and adaptive immune systems, are potential targets for vaccine adjuvants, and have received increased attention from the scientific community. PolyI:C is a TLR3 ligand that mimics viral dsRNA and is a promising immunostimulant candidate for vaccines directed against intracellular pathogens (9). PolyI:C induces innate immune response similar to a live viral vaccine (10), interferon-alpha/beta (IFN-α/β) production, and a stable maturation of human MoDCs and CD8^+^ T cells immune response (11). AddaVax and PolyI:C have present promising results for influenza vaccine development (12–16). Mice firstly immunized with antigen emulsified in AddaVax adjuvant, and then boosted with antigen combined with AddaVax and TLR9 agonist CpG-DNA, showed a higher titer response (17) In addition, AddaVax shows antigen delivery effect (18), implying that AddaVax can be used as a carrier for other adjuvants. PolyI: C can be used as a component of a novel adjuvant (19). Previous studies report that combining TLR agonists and w/o adjuvants can induce higher immune antibody titers and cellular response, thus can be used as potential adjuvants.

AP comprising PolyI:C formulated with AddaVax may be able to be used as influenza vaccine adjuvant based on the immune enhancement effects of w/o adjuvants and TLR agonists. The purpose of this research is to strengthen immunology research, further improve the understanding of acting mechanisms of adjuvant and fuel the new combination adjuvant**.** MDCK-based inactivated influenza vaccine was combined with AP, AddaVax, or PolyI:C. Antibody titers and safety were evaluated after intramuscular (i.m.) at different injection schedules. The local innate response, humoral and cellular responses induced by MDCK-based influenza vaccine with and without AP adjuvant were evaluated in a mice model.

## MATERIALS AND METHODS

### Ethics Statement

Animal studies were approved by the medical ethics committee of Wuhan Institute of Biological Products (WIBP-AII312,020,001). All experiments were performed following the relevant guidelines and regulations of Laboratory Animal Guidelines for Ethical Review of Animal Welfare (Standardization Administration of China. 2018) (20).

#### Vaccine Formulations and Adjuvants

Influenza H3N2 vaccine strain was purchased from National Institute for Biological Standards and Control (NIBSC) according to seasonal influenza vaccine strains recommendation by World Health Organization. H3N2 vaccine strain: Influenza Reagent Influenza Virus Infectious IVR-195 was used in the current study.

The H3N2 (H3) vaccine strain used in the current study was propagated in MDCK cells (MDCK CCL-34, ATCC). Hemagglutinin (HA) antigen content in vaccine higher than 120 μg/mL determined by a single radial immunodiffusion (SRID) assay was selected. AddaVax (InvivoGen, San Diego, CA), polyinosinic-polycytidylic acid (PolyI:C, Sigma, USA), AP (PolyI:C and AddaVax combined adjuvant) were used in this study.

#### Mouse Immunizations

Female BALB/c mice (6–8 weeks old, about 18–20 g in body weight) or Wistar rats with weight of 175–200 g were provided by Animal Experimental Center of Wuhan Institute of Biological Products Co. Ltd. All animals were injected intramuscularly which is similar to the injection route of vaccines in humans. Capillary tube was used for collection of blood from the venous sinus of eyes.

#### Evaluation of the Immunogenicity of AP-Adjuvant Vaccine

To assess the immunogenicity of AP-adjuvanted influenza inactivated vaccine, 90 BALB/c mice or Wistar rats were divided to 9 groups, each with 10 animals. The animals were immunized with H3 alone (15 μg, 9 μg, 6 μg, and 3 μg/mouse) or adjuvanted with AP (1:1, vol:vol ratio, the concentration of PolyI:C in AP was 50 μg/mouse). PBS was used as a negative control. The value of H3-specific hemagglutination inhibition (HI) titer was measured 21 days after the first injection of the aforementioned agents.

To determine the long-term effectiveness of the antibody, BALB/c mice received H3 (5 μg/mouse) alone or in combination with AddaVax (1:1, vol:vol ratio), PolyI:C (50 μg/mouse), or AP (1:1, vol:vol ratio); two doses were given at a 3-week interval. The HI titer was measured on day 21, 100, 200, and 270 after the booster immunization. Blood samples were collected 5 days after booster immunization to measure levels of H3-specific total IgG, IgG2a, and IgG1.

#### HI Assay

Prior to HI testing, fresh chicken erythrocytes were washed with phosphate-buffered saline (PBS, pH 7.4) for three times in 15 mL centrifuge tube at 1500 rpm for 5 min; then, packed chicken erythrocytes and 1.0% suspension of chicken erythrocytes were prepared in PBS. All serum samples were pre-treated with receptor-destroying enzyme (RDE) (Sigma-Aldrich, USA) for 18 h at 37 °C to prevent nonspecific inhibition, then treated with packed chicken erythrocytes for 1 h at 4 °C to remove natural serum agglutinins. The serum samples were twofold diluted with PBS serially in a 96-well V-bottom plate (with an initial dilution of 1:5). A fixed concentration of standardized influenza virus HA antigen (4 HAU/25 μL) was added to each well to allow binding between HA and serum anti-HA antibodies. The serum–virus mixture was incubated at room temperature for 40 min. Furthermore, 1% erythrocytes (25 μL/well) were added and allowed to settle at room temperature for 30 min. Antibody titer was expressed as the reciprocal of the highest serum dilution that showed complete inhibition of hemagglutination. The starting dilution was 1:5; thus, the lowest detection threshold was 5.

#### H3-Specific Immunoglobulin Subclasses

Blank enzyme-linked immunosorbent assay (ELISA) plates were coated with H3 vaccine (3 μg/mL, 100 μL/well) overnight at 4 °C. Plates were washed three times for at least 1 min with PBS-T (**PBS** with 0.05% Tween 20, pH 7.4). Plates were blocked with 200 μL/well of 1% bovine serum albumin (BSA) (China National Pharmaceutical Group Co., Ltd, China) for 1 h; then, plates were washed. Serum was tested at a starting dilution of 1:100. After incubation for 1 h at 37 °C, plates were washed and incubated with 1:50,000 diluted goat anti-mouse IgG conjugated to horseradish peroxidase (HRP) (Bethyl, China), goat anti-mouse IgG1 conjugated to HRP (Bethyl, China), or 1:5000 diluted goat anti-mouse IgG2a conjugated to HRP (bersee, China) at 37 °C for 1 h. The 3,3′,5,5″-tetramethylbenzidine (TMB) substrate set (Biolegend, CA, USA) was added for color reaction. 1 mol H_2_SO_4_ was added to stop the color development after 5 min. Optical density (OD) was determined at 450 nm using a Multiskan FC® microplate photometerm (Thermo Fisher Scientific, USA). The titers of IgG, IgG1, or IgG2a were expressed as the last dilution that showed > 0.1 absorbance over background levels.

#### Antigen-Specific Splenocyte Stimulation

BALB/c mice were immunized i.m. with H3, H3 + Add, H3 + PolyI:C, and H3 + AP (5 μg/mouse) twice at 3-week intervals; 5 days after the second immunization, four spleens representing one group were used for preparing cell suspension. Cells were cultured in 24-cell culture plates containing 1640 medium (Sangon Biotech, China) and 10% FBS, and re-stimulated with H3 antigen (1 or 5 μg/mL) for 3 days. The levels of cytokines (IL-1β, IL-4, IL-5, IL-6, IL-10, IL-13, and IFN-γ) in the supernatant were measured with cytometric bead array (CBA) kit (Biolegend, CA, USA) using a CytoFLEX LX Flow Cytometer (Beckman Coulter, USA).

#### Analysis of Antigen-Specific Immune Cells

BALB/c mice were immunized twice at an interval of 3 weeks with H3, H3 + Add, H3 + PolyI:C, and H3 + AP (5 μg/mouse); 5 days after the booster immunization, the number of activated IL-4- and IFN-γ-secreting immune cells was quantified using the enzyme-linked immunospot (ELISpot). Fresh mouse splenocytes were isolated and added at 2 × 10^5^ /well in mouse IFN-γ precoated ELISpot plate (Dakewe Biotech Co., Ltd., China) or mouse IL-4 precoated ELISpot plate (Dakewe Biotech Co., Ltd., China). H3 antigen (5 μg/mL) as stimulus was added into each well. Splenocytes with 1 × 10^5^ /well were stimulated with PMA (phorbol myristate acetate) (10 ng/well) as the positive-control group. The results were detected after 24 h with ELISpot kit following the manufacturer’s protocols.

#### Spatiotemporal Consistency of H3 and AP

To further explore the adjuvant activity of AP in inducing cellular and antibody reponse, BALB/c mice (*n* = 10/group) were injected with AP and H3 (5 μg/mouse) at the same injection site with intervals of 0, 1, 24, and 48 h (AP and H3 was administered using different syringes) respectively; AP and H3 (5 μg/mouse) was pre-mixed and injected in one syringe as control group, and AP and H3 (5 μg/mouse) was injected separately at contralateral hind thigh muscles (remote sites) simultaneously as another group to evaluate effect of different injection sites on the antibody response induced by H3 and AP.

#### Cytokines and Chemokines Detection in the Muscle Injection Site

BALB/c mice(*n* = 4/group) received H3 and H3 + AP (5 μg/mouse) through intramascular route and muscles at the injection site were harvested at different time points from 0, 24, 48, and 120 h. Muscles were seperately homogenized in 1 mL PBS; then, the homogenates were cleared by centrifugation. Protein levels in the cleared homogenates were determined by CBA kit (Biolegend, CA, USA).

#### Analysis of Cytokine and Chemokine mRNA Levels in the Muscle Injection Site

##### mRNA Extraction

BALB/c mice (*n* = 3/group) were first injected with 100 μL AP, or PBS in thigh muscles and sacrificed at 4 and 24 h after injection. The muscle of the injected site was removed and stored in RNAlater TM (Invitrogen, USA) for further processing. Tissues were homogenized in Motor-Driven Tissue Grinder (Sangon Biotech, China); total RNA was extracted using RNeasy TM Minikit (Qiagen, Germany). DNAse treatment was performed on RNeasy TM column to avoid genomic DNA contamination. The concentration of RNA was quantified by microdrop (Thermo Fisher Scientific, USA).

##### qRT-PCR

About 50–500 ng of total RNA was reverse-transcribed with Oligod T primers. cDNA was then diluted three times. Real-time PCR mix was 20 μL in volume and was prepared according to instructions of the One Signal Pathway Detection SYBR Green Real-Time RT-PCR Superkit (Heifei Zhi En Biology, China). All specific primers are listed in Table I. The mix was subjected to real-time PCR analysis on ABI 7500 Fast Real-Time PCR System (Thermo Fisher Scientific, USA). All analyses were repeated three times, and outliers were excluded from analysis. The standard Ct values were normalized based on DAPDH as the endogenous control. Each gene expression was determined as the ratio of log2 transformed RT-PCR measurement for AP group in muscle relative to the mean value of matched PBS-treated controls.

**Table I:** Cytokine and Chemokine Primers for RT-PCR

Gene name	Primer sequence 5′-3′	Length
IL-6	F 5′-CTTCTTGGGACTGATGCTGGT-3′ R 5′-AGACAGGTCTGTTGGGAGTGG-3′	98
INF-α	F 5′-GGCTCCCAGTGGAAGCAAA-3′ R 5′-CTCTTCTCGATGGCACCCTT-3′	103
INF-β	F 5′-TGACGTGGGAGATGTCCTCAA-3′ R 5′-ACCATCCAGGCGTAGCTGTT-3′	103
INF-γ	F 5′-CAGGCCATCAGCAACAACATAA-3′ R 5′-GGCAATACTCATGAATGCATCC-3′	99
CCL2	F 5′-ATGCAGTTAACGCCCCACTC-3′ R 5′-CTGCTGCTGGTGATCCTCTT-3′	97
CCL3	F 5′-CGGAAGATTCCACGCCAATTC-3′ R 5′-TCTTTGGAGTCAGCGCAGAT-3′	116
CCL4	F 5′-CCAGGGTTCTCAGCACCAAT-3′ R 5′-TCTGCCTCTTTTGGTCAGGA-3′	156
CXCL1	F 5′-CCCAAACCGAAGTCATAGCC-3′ R 5′-ACTTGGGGACACCTTTTAGCA-3′	111
CXCL2	F 5′-CCAGTGAACTGCGCTGTCAA-3′ R 5′-GGGCGTCACACTCAAGCTCT-3′	80
CXCL3	F 5′-AGTGAGCTGCGCTGTCAGT-3′ R 5′-TTCTGTCTGGGTGCAGTGG-3′	105
CXCL5	F 5′-GAGCTGCGTTGTGTTTGCTT-3′ R 5′-GCTATGACTTCCACCGTAGGG-3′	110

*F*, forward primer; *R*, reverse primer

The primer sequences used for the detection of cytokines and chemokines were obtained from NCBI

#### DCs Activation and T Cell Priming

Immature bone marrow dendritic cells (BMDCs) were generated with RPMI-1640 (Sangon Biotch, China) with granulocyte–macrophage colony-stimulating factor (GM-CSF) (Cyagen, China) as previously described (21). The immature BMDCs were then incubated with AddaVax (5 μL), PolyI:C (5 μg), AP (AddaVax 5 μL and PolyI:C 5 μg) in 24-well plates in 1 mL complete media at 37 ℃. After incubation for 24 h, BMDCs were harvested, washed, and stained with Brilliant Violet 605 anti-mouse CD11c, PerCP/Cy5.5 H-2Kd/H -2Dd, PE CD40, APC anti-mouse CD80, PE/Cy7 anti-mouse CD86 (Biolegend, USA). Costimulatory factors on BMDCs including MHCII, CD40, CD80, and CD86 were analyzed by flow cytometry. IL-12p70 secreted from BMDCs in cell culture medium was detected by ELISA kits (Abcam, USA).

To explore the antigen uptake by BMDCs, BMDCs suspension was seeded on confocal petri dish coated with poly-l-lysine (Sangon Biotech, China), and incubated with Cy5.5-labeled H3 (Cy5.5-H3, 5 μg/mL) formulated with AP (5 μL/mL AddaVax and 5 μg/mL PolyI:C), AddaVax (5 μg/mL), or PolyI:C (5 μg/mL) for 6 h. Meanwhile, BMDCs suspension was incubated with FITC-labeled PolyI:C (FITC-PolyI:C, 5 μg/mL) with or without AddaVax (5 μg/mL) as other groups. Cell nuclei were stained with 4’,6-diamidino-2-phenylindole (DAPI) (Beyotime, China). Cy5.5-H3 and FITC-PolyI:C uptake by BMDCs was observed under a Nikon C2-ER laser confocal microscope (Nikon Corporation, Japan).

Five days after the booster injection with H3, H3 + AP, H3 + Add, H3 + PolyI:C (5 μg/mouse), CD4^+^ T cells from the spleens of mice were isolated by flow sorting. Co-incubated BMDCs and CD4^+^ T cells (1 × 10^5^ each) were seeded in 96 well cell culture plate treated with H3, H3 + AP, H3 + Add, H3 + PolyI:C (5 μg/mL) respectively. The levels of IL-4 and IFN-γ within the supernatant were determined using ELISA kits (Dakewe Biotech Co., Ltd., China) after 48 h co-cultivation..

#### Tracking Migration of H3 to LNs

BALB/c mice (*n* = 3/group) received Cy5.5-H3, Cy5.5-H3 + AP (5 μg/mouse) by intramuscular injection. Four consecutive frozen sections of draining LNs were prepared at 48 h after immunization. The location of Cy5.5-H3 and immune cells in LNs was detected by immunofluorescence. Monocytes and macrophages were defined as CD11b, T cells as CD3, B cells as B220, DCs as CD11c.

#### Phenotyping of LNs Cells by Flow Cytometry

BALB/c (*n* = 15/group) mice were first injected with H3, H3 + Add, H3 + PolyI:C, H3 + AP (5 μg/mouse) in thigh muscle, then sacrificed at 4, 24, and 60 h post injection. Five LNs cells were pooled, then homogenized and treated with TruStain FcX anti-mouse CD16/32 to block Fc receptor. Three pools represented a group. Monocytes were defined as Cd11b^+^, F4/80^+^ cells; DCs as CD11b^−^, CD11c^+^, MHCII^+^; granulocyte as Ly6G^+^, CD11b^+^; T4/T8cells as CD3^+^CD4^+^/CD8^+^ cells; B cells as CD3^–^, CD19^+^.

#### Tracking Local Migration and Metabolism of H3 using *In Vivo* Fluorescence Imaging

To confirm the migration and metabolism of H3 in BALB/c mice, mice were injected with Cy5.5-H3, Cy5.5-H3 + Add, Cy5.5-H3 + PolyI:C, Cy5.5-H3 + AP (5 μg/mouse) in thigh muscle. Bioluminescence images were acquired on day 0, 2, 5, 7, 9, 11, 13, and 15 after immunization. The mice were anesthetized with 4% isoflurane exposure; then, 670 nm irradiation was implemented to the whole mouse for fluorescence images using PE IVIS Lumina XRMS (PerkinElmer, USA).

#### Safety of AP-Adjuvanted H3 Vaccine

Female BALB/c mice were administered with H3, H3 + Add, H3 + PolyI:C, H3 + AP (10 μg/mouse). Three days after the first injection, the size and texture of draining LNs were detected and graded as previously described (22). For determination of spleen index, the spleens were isolated and weighted 5 days after booster injection.

Female BALB/c mice and Wistar rats were randomized into 5 groups and administered with 45 μg H3, 500 μL AP, 15 μg H3 + AP, 45 μg H3 + AP, and 500 μL PBS. To explore safety of AP and H3 vaccine, three injection doses were performed at 2-week intervals, and body weight and vital signs were recorded from day 1 to day 7 and on day 14 after each injection. Levels of C-reaction protein (CRP) in serum as inflammatory marker were determined on day 7 after the last injection with ELISA kit (Abracon, China) for Wistar rats and ELISA kit (Shanghai Guangrui Biological Technology Co., Ltd, China) for BALB/c mice.

#### Statistical Analysis

All statistical analyses were performed using the GraphPad Prism 9.00 software (GraphPad Software Inc., CA, USA). Serum HI and IgG titers were analyzed by logarithmic transformation method. Data normality was confirmed using Anderson-Daring, Shapiro–Wilk test, and Kolmogorov–Smirnov test. One-way or two-way analysis of variance (ANOVA), Dunnett *t* test, or Kruskal–Wallis tests were performed based on normal distribution of data and homogeneity of variance. Statistical significance was assigned when *p* ≤ 0.05, and represented as*p ≤ 0.05, **p < 0.01, and ***p < 0.001 in figures.

## RESULTS

### The Immunogenicity of H3 Adjuvanted with AP

**Fig. 1:**
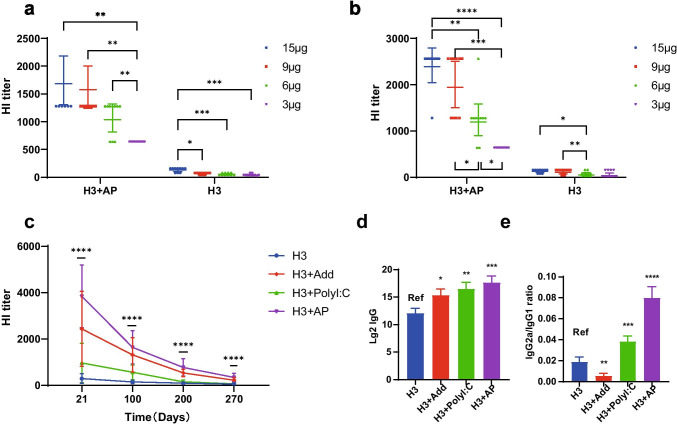
AP improved the antibody response. HI titers were analyzed on day 21 after immunization of BALB/c mice **a** and Wistar rats **b** with 3 μg, 6 μg, 9 μg, 15 μg H3 combined with AP. For long-lasting immunity after the boost-immunization, mice were injected with 5 μg H3, H3 + AP, H3 + Add, and H3 + PolyI:C; serum HI titers were analyzed on day 21, 100, 200, and 270 **c**. H3-specific IgG **d**, isotype antibody ratios (IgG2a/IgG1) **e** were calculated based on ELISA results, geometric mean titer (GMT) ± CI were used to represent HI titers and IgG levels, mean ± CI was presented for IgG2a/IgG1. Statistical comparisons were performed using two-way ANOVA for **a**, **b**, **c**. Kruskal–Wallis test was used for **d**, **e**. *P < 0.05; **P < 0.001, P*** < 0.001, ****P < 0.0001; ns, not significant. Note: Ref represents the reference group

The HI test is the most commonly used serological assay to detect influenza specific antibodies titer. The geometric mean titer (GMT) of HI antibody to H3 vaccine strain of BALB/c mice immunized with 15 μg, 9 μg, 6 μg, or 3 μg/mouse H3 with or without AP was 1689, 1575, 1039, 640 and 129, 64, 52, 40 respectively, indicating dose-dependent vaccine effects (Fig. 1a). In different species annimals, such as Wistar rats, the dose-dependent effects were also significant in H3 + AP and H3 groups (Fig. 1b). AP improved the immunogenicity of H3, because at the same dose, HI titer in H3 + AP group was higher compared with that in H3 group (Fig. 1a, b).

For long-term immunogenicity evaluation, HI titer among H3, H3 + Add, H3 + PolyI:C, H3 + AP groups decreased with time (Fig. 1c). HI titer detected in different groups showed significant differences at day 21, 100, 200, and 270 after the booster immunization. On day 270 after the booster immunization, the HI titer of H3 + AP group was at a relatively higher level (HI titer > 640) compared with that of H3 group with only 50% of mice having antibody titer over 40**,** implying that AP significantly induced long-term humoral response with a high level of antigen-specific antibodies.

Advantages of ELISA in detection of influenza antibodies include its ability to determine different class-specific IgG2a, IgG1, IgG antibodies in serum samples and repeatability. H3 + AP induced significant amounts of H3-specific IgG when compared to H3 injected mice (Fig. 1d), which was consistent with the results of HI titers. Th1 immune response induces IgG2a production, whereas Th2 induces production of IgG1 type antibodies. The increased IgG2a/IgG1 ratio can be used to evaluate vaccine-induced cellular-based immune responses. The ratio of IgG2a/IgG1 induced by H3 + AP was higher compared with that induced by H3 + Add, H3 + PolyI:C or non-adjuvanted H3 (Fig. 1e).

### AP Adjuvanted H3 Elicited Cellular and Humoral Immune Response in Mice

**Fig. 2:**
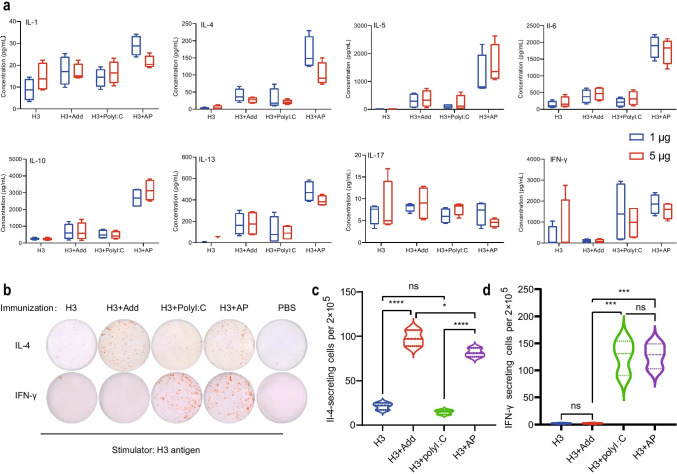
Th1 and Th2 cytokines and cells induced by AP. Cytokines secreted by splenocytes under the stimulation of H3 (1 or 5 μg) in immunized mice at 5 day after booster injection **a**. Results are presented as mean (min,max). ELISpot analysis of IL-4 and IFN-γ secreting cells after restimulated with H3 antigen after the booster immunization **b**, **c**, **d**. Dunnett *t* test was used for statistical analysis. *, significant; ns, not significant

To further elaborate the types of immune response enhanced by AP, splenocytes of mice injected with H3 + AP and H3 were stimulated with H3 antigen (1 or 5 μg/mL). No differences were obtained in the cytokine secretion induced by splenocytes stimulated with 1 and 5 μg/mL H3 antigen. Splenocytes of mice injected with H3 + AP secreted higher level of IFN-γ, IL-4, IL-5, IL-6, IL-10, and IL-13 compared with those injected with H3 alone (Fig. 2a). IFN-γ is related to Th1 cellular immunity, whereas IL-4, IL-5, IL-6, IL-10, and IL-13 are associated with Th2 response.

Activated immune cells were implicated in production of cytokines. Number of IFN-γ and IL-4 producing cells was determined by ELISpot. The number of IFN-γ or IL-4 inducing cells in H3 + Add, H3 + PolyI:C, and H3 group was compared with those in H3 + AP group. The findings showed no difference in the number of IL-4 secreting cells between H3 + Add and H3 + AP group, indicating that AddaVax in AP induced the activation of IL-4 secreting cells. In addition, analysis showed no difference in level of IFN-γ secreting cells between H3 + AP and H3 + PolyI:C group (Fig. 2b, c, d), indicating that PolyI:C in AP induced the activation of IFN-γ secreting cells. In summary, these findings demonstrate that AP enhances both cellular and antibody mediated immunity.

### The High Antibody Titer Induced by H3 + AP Depends on H3 and AP Injection Site and Time

**Fig. 3:**
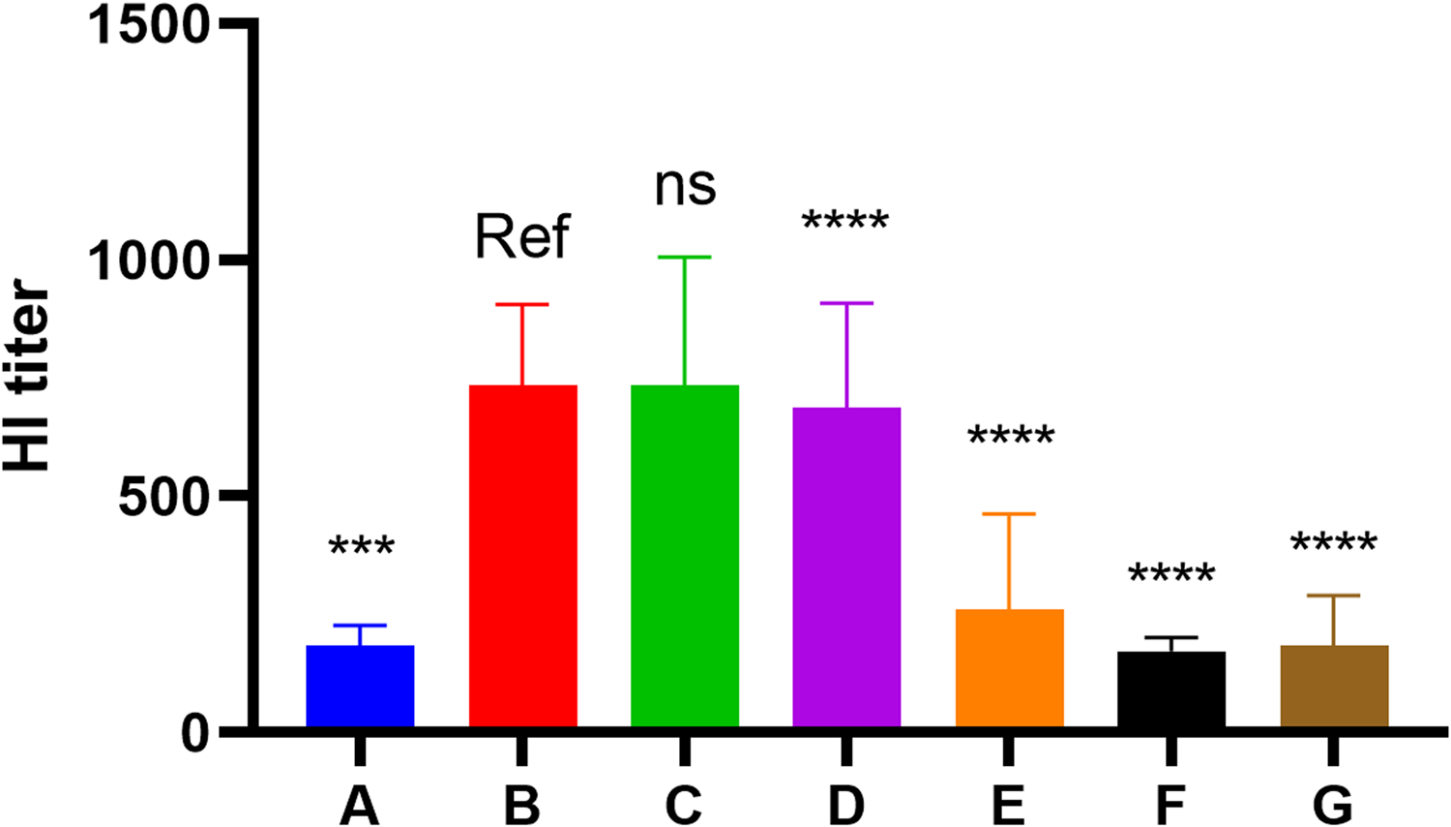
Spatio-temporal co-localisation of AP and H3. BALB/c mice (10 per group) were injected with 5 μg H3 and AP at different times (0–48 h), A group: H3 injected alone; B group: H3 and AP were pre-mixed before injected; C group: H3 and AP was injected using two syringes separately at the same site and same time without pre-mix. D, E, F group: H3 and AP injected at the same site with 1, 24, and 48 h intervals respectively, G group: H3 was injected on the left leg thigh muscle but AP was injected on the right leg muscle. HI titer was analyzed on day 21 after injection. All other groups were compared with B group using Kruskal–Wallis test; *, significant; ns, not significant. Note: Ref represents the reference group

HI titer of mice injected with pre-mixed H3 + AP was not higher compared with those injected with AP and H3 separately at the same injection site with two syringes without premixing (Fig. 3). The HI titer of the group injected with H3 and AP at contralateral thigh muscles was similar with the HI titer of mice administered with H3 alone. Administration of pre-mixed H3 + AP showed significantly higher HI titer compared with the HI titer of mice injected with AP and H3 at different injection intervals at same injection sites. An increase in injection interval between AP and H3 was correlated with a decrease in HI titer (Fig. 3). These findings showed that adjuvant activity is not dependent on the physical association with the antigen, but on the injection site and time, further demonstrate that AP adjuvant activity in inducing higher HI titer was transient and local to the injection site may be related to the AP-induced local immune microenvironment.

### AP Induces Production of Cytokines at Injection Site

**Fig. 4:**
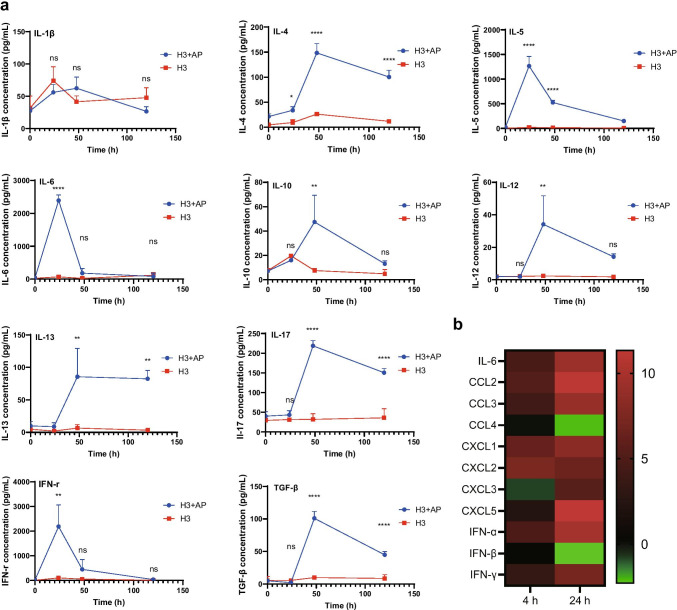
Cytokines and chemokines were upregulated by AP in the local injection environment. BALB/c mice (*n* = 4/group) were administered with H3 + AP or H3 alone. The muscle of the injected site was homogenated before or at 24, 48, and 120 h after injection. **a** Levels of IL-1β, IL-4, IL-5, IL-6, IL-10, IL-12, IL-13, IL-17, IFN-γ, and TGF-β were determined by CBA. **b** Local mRNA expression levels of cytokine and chemokine after immunization. BALB/c mice were administered with AP or PBS, and mRNA expression of IL-6, CCL2, CCL3, CCL4, CXCL1, CXCL2, CXCL3, CXCL5, IFN-α, IFN-β, IFN-γ at the injection site of muscle were determined at 4 and 24 h by qRT-PCR. mRNA expression levels of the cytokines were normalized using DAPDH as endogenous control. Two-way ANOVA was used. *, significant; ns, not significant

To explore the change in immune microenvironment induced by AP, the levels of cytokines at the injection site were compared after the injection of H3 + AP or H3 alone at 0, 24, 48, and 120 h. Pro-inflammatory cytokines IL-5, IL-6 and macrophage activating factor IFN-γ induced by H3 + AP reached peak at around 24 h after injection, and were 20- to 60-fold higher compared with the levels induced by H3 alone. Levels of IL-10 and transforming growth factor-beta (TGF-β), IL-4, IL-13, IL-17, and IL-12 induced by H3 + AP reached peak at around 48 h after injection and were 5- to 15-fold higher compared with the levels induced by H3 alone. Detected cytokines at the intramuscular injection site of mice induced by H3 alone showed low levels, as well as IL-1β induced by H3 + AP and H3 (Fig. 4a). Induction kinetics of cytokines showed that H3 + AP exhibited high efficacy in inducing immune microenvironment compared with H3 alone. Peak levels of cytokines were consistent with adjuvant activity (within 48 h), which means that protective antibodies induced by H3 + AP were implicated in AP induced innate immunity.

mRNA expression levels of IL-6, IFN-α, IFN-γ CCL2, CCL3, CXCL1, CXCL2, CXCL3, CXCL5 were significantly higher (4- to tenfold) after administration of AP compared with PBS at 4 and 24 h in the muscle (Fig. 4b). mRNA expression levels of IFN-γ and IL-6 after administration of AP were consistent with levels of cytokines induced by H3 + AP. The chemokines and inflammatory factors were implicated in immune cell recruitment, activation and expression at the injection site, mainly the antigen-presenting cells (APC).

### AP Promotes Migration of Antigen and Immune Cells in LNs

**Fig. 5:**
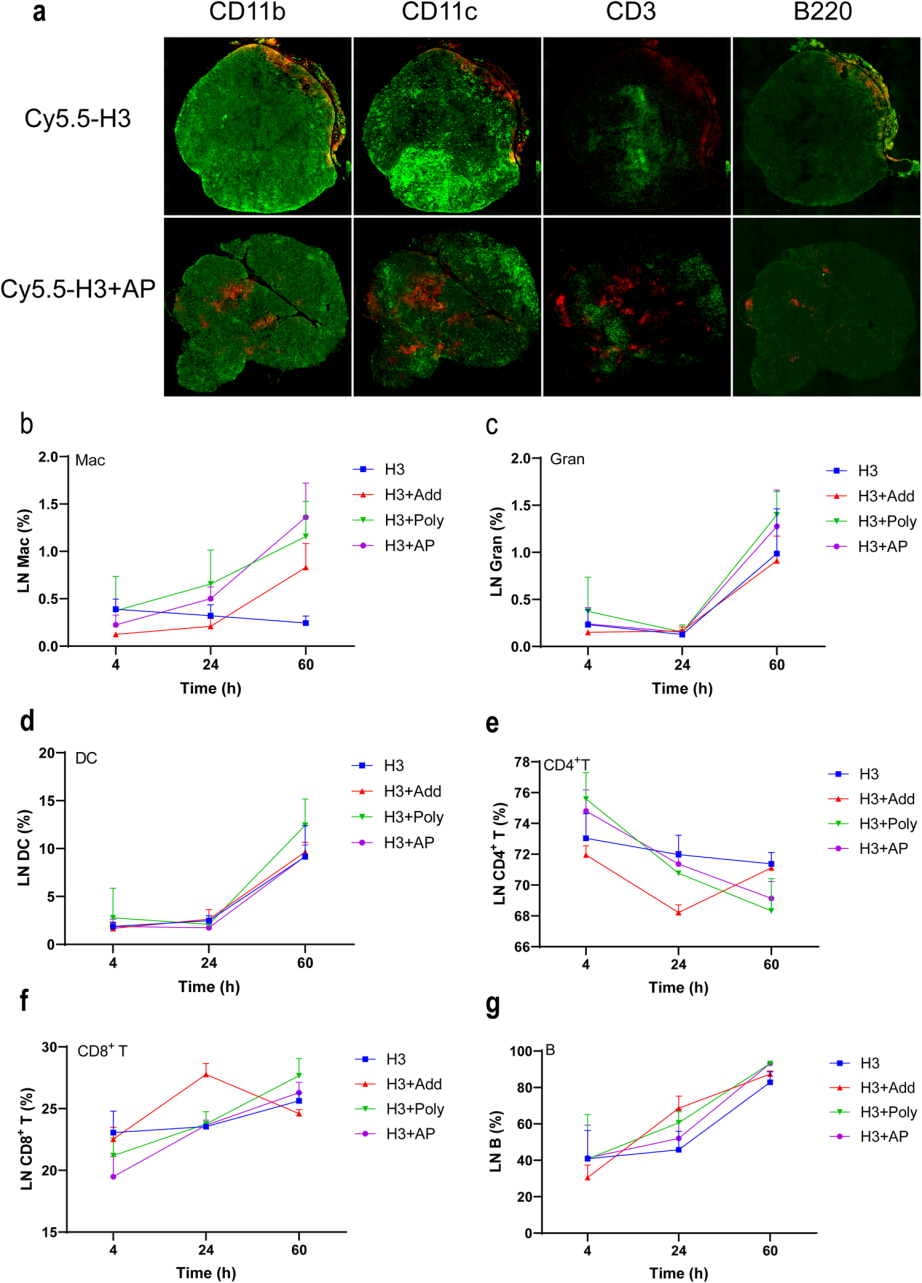
H3 migration and immune cells proliferation in LNs. Cy5.5-H3 (5 μg, 100 μL/mouse) was injected into BALB/c mice i.m. with AP, AddaVax, PolyI:C. Forty-eight hours later, draining LNs were isolated and 4 frozen sections were made by serial sectioning. Green fluorescence represents CD11b, CD11c, CD3, B220 labeled cells and red represents Cy5.5-H3 **a**. Original magnification, × 10. BALB/c mice (*n* = 15/group), five LNs form a pool; each pool of LNs represents a date. Mice were injected with 5 μg H3 with AP, AddaVax, PolyI:C, or H3 alone; LNs were obtained at 4, 24, and 60 h after injection. The number of macrophages **b**, granulocytes **c**, DCs **d**, T cells **e**, **f**, and B cells **g** was determined by flow cytometry. Data is presented as mean ± SD and two-way ANOVA was used. ns means not significant, * means significant for at least one group at the same point in time

Fluorescence of Cy5.5-H3 adjuvanted AP was observed at the center of LNs 48 h after administration; however, fluorescence of Cy5.5-H3 alone was not easily detected at the center of LNs (Fig. 5a), indicating that AP promotes migration of H3 antigens to LNs. T cells, B cells, macrophages, and DCs presented in the draining LNs were detected. Number of macrophages, mature DCs, and granulocytes induced by H3, H3 + AP, and H3 + PolyI:C at 60 h after injection were 2- to threefold more compared with that induced at 4 h, except for macrophages induced by H3 (Fig. 5b, c, d). The number of immune cells in LNs did not change significantly from 4 to 24 h. H3 + Add induced a decrease in the number of CD4^+^ T cells but an increase in CD8^+^ T cells which was correlated with cellular response (Fig. 5e, f). H3 + AP, H3 + PolyI:C, and H3 + Add induced a larger number of B cells at 60 h in LNs (Fig. 5g). In summary, AP induces H3 antigen uptake by DCs and macrophages, and enhances the progression of local immunity to adaptive immunity.

### AP Induces DCs Activation and Antigen Presentation

**Fig. 6:**
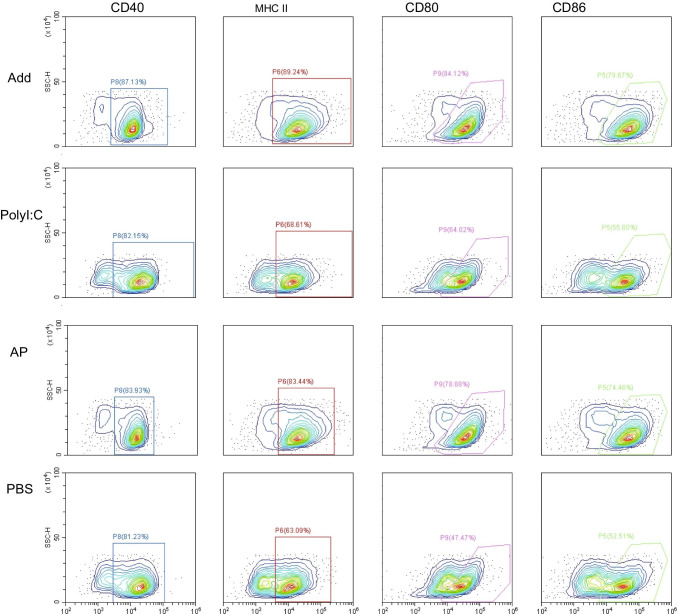
The maturation marker expression on the surfaces of BMDCs. BMDCs (2 × 10^6^ cells) were stimulated with AP (5 μL/mL AddaVax and 5 μg/mL PolyI:C), AddaVax (5 μL/mL), or PolyI:C (5 μg/mL) for 24 h. DCs maturation markers were assessed based on CD86, CD40, CD80, and MHCII expression levels through flow cytometry

**Fig. 7:**
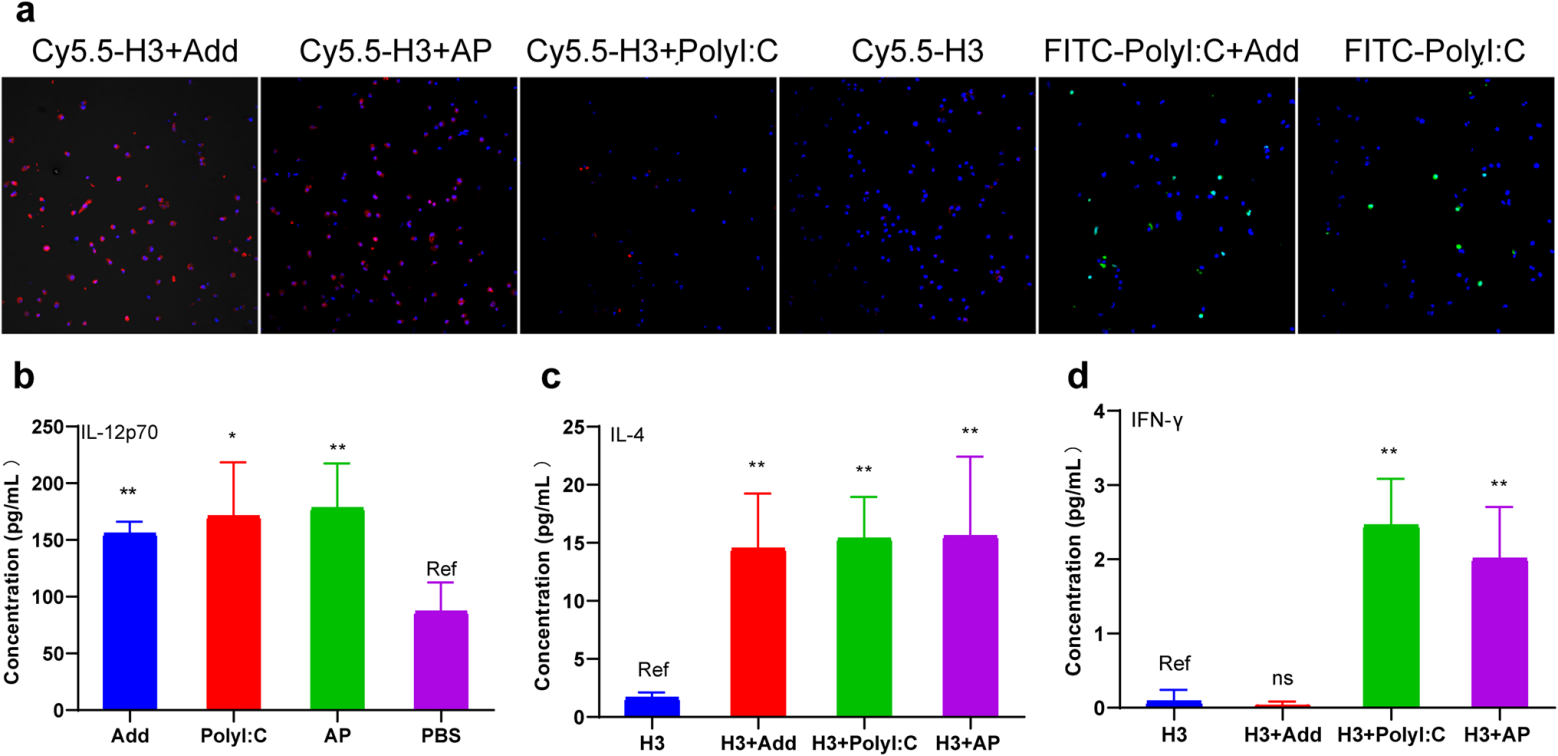
AP promotes differentiation of DCs and T cells. **a** DCs uptake of antigen, BMDCs were stimulated with 5 μg/mL Cy5.5-H3 mixed with AP, AddaVax, PolyI:C or 5 μg/mL FITC-PolyI:C with or without AddaVax. **b** IL-12p70 secreted by BMDCs was analyzed 24 h after stimulation with AP, AddaVax, and PolyI:C. T cells were sorted from splenocytes of mice injected with 5 μg H3, H3 + AP, H3 + Add, and H3 + PolyI:C. BMDCs cultured with T cells and stimulated with different formulations (5 μg/mL H3, H3 + AP, H3 + Add, H3 + PolyI:C). The levels of IL-4 **c** and IFN-γ **d** were analyzed 48 h after co-culture. Data are expressed as mean ± SD (*P < 0.05; **P < 0.001, P*** < 0.001, Dunnett *t* test was used). Ref represents the reference group

DCs play a role in presenting antigens to lymphocytes and initiate adaptive immune response. Cell-surface expression of MHCII, CD40, CD80, and CD86 on BMDCs was significantly increased after stimulation with AddaVax and AP compared with administration of PolyI:C or PBS (Fig. 6). High red fluorescence intensity showed that Cy5.5-H3 was concentrated within BMDCs cytoplasm, when BMDCs were incubated with AP or AddaVax formulated with Cy5.5-H3. However, red fluorescence was difficult to observe in the center of LNs when BMDCs were cultured with Cy5.5-H3 alone. PolyI:C did not significantly induce uptake of antigen compared with AP. In addition, AddaVax did not significantly increase FITC-PolyI:C (blue) uptake compared with FITC-PolyI:C alone (Fig. 7a). These results confirmed that AP is effective in mediating intracellular transfer of protein antigens but not double-stranded RNA.

IL-12, known as IL-12p70, is mainly produced by activated inflammatory cells. DCs are the first cells to synthesize IL-12. AP, AddaVax, and PolyI:C significantly promoted release of IL-12p70 from BMDCs compared with PBS (Fig. 7b). Mouse BMDCs were co-cultured with CD4^+^ T cells extracted from mouse spleen after immunization for 48 h. The findings showed that H3 + Add, H3 + PolyI:C, and H3 + AP significantly increased secretion of IL-4 from co-cultured BMDCs and T cells. Moreover, H3 + PolyI:C and H3 + AP promoted secretion of IFN-γ from co-cultured BMDCs and T cells (Fig. 7c, d). These findings indicated that AP promotes effective activation, costimulatory factors expression, and cytokine secretion of DCs. In addition, AP promotes uptake of antigen and interaction between DCs and T cells, thus inducing Th1 and Th2 response.

### AP Promotes Metabolism of H3 in the Body

**Fig. 8:**
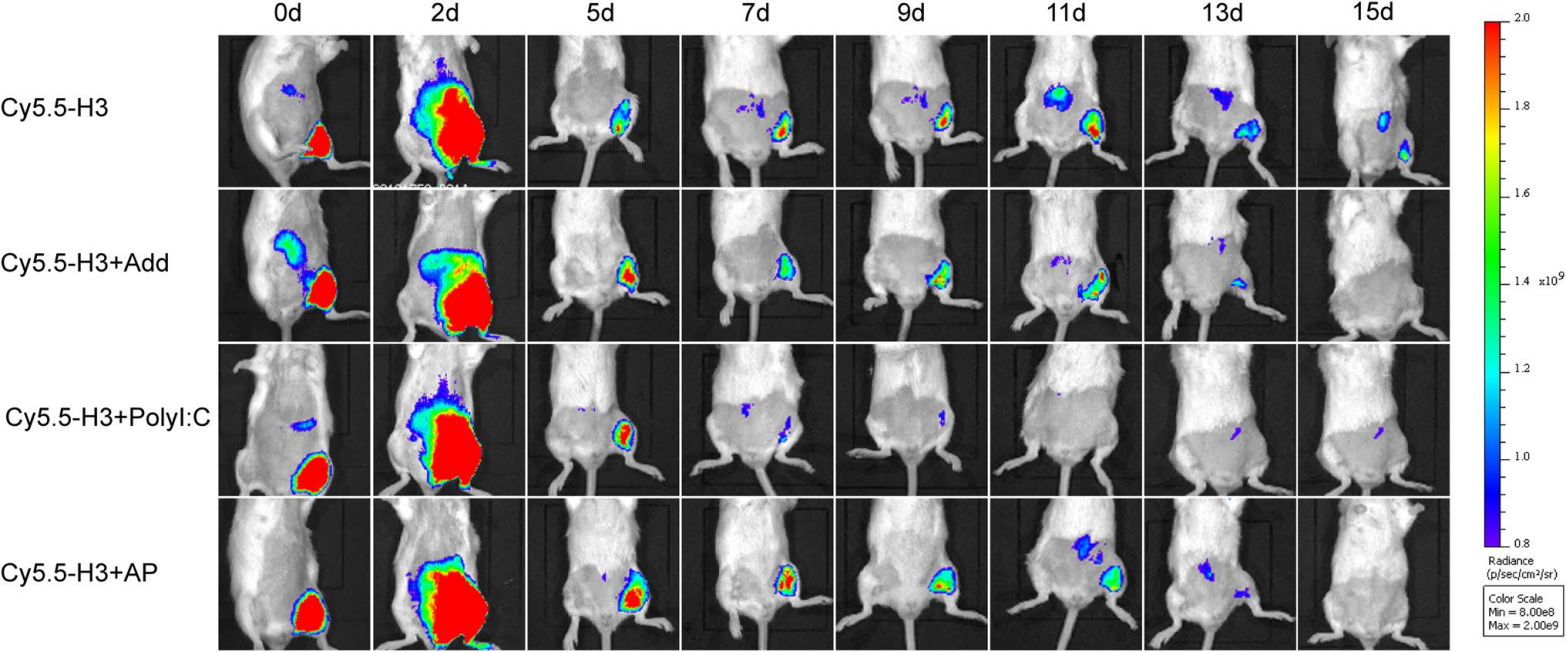
Fluorescence imaging after administration of H3 combined with adjuvant in mice. Cy5.5-H3 alone or with AP, PolyI:C, and AddaVax were injected into BALB/c mice (100 µL/mouse), and fluorescence images were acquired using PE IVIS Lumina XRMS. Real-time monitoring of H3 antigen persistence at the injection sites or migration by *in vivo* fluorescence imaging

To explore effects of AP on migration and metabolism of H3, mice were vaccinated with Cy5.5-H3 alone or with AP through intramascular route. Bioluminescence images were acquired on day 0, 2, 5, 7, 9, 11, 13, and 15 after vaccination. The findings showed that AP promoted metabolism of H3 (Fig. 8). On day 2 after immunization, the fluorescence was the highest at the immune site, whereas fluorescence decreased over time. Compared with mice injected with Cy5.5-H3, fluorescence decreased faster in mice treated with Cy5.5-H3 + AP, Cy5.5-H3 + Add, Cy5.5-H3 + Poly:C. The fluorescence of Cy5.5-H3 in mice was difficult to detect on day 11 of H3 + PolyI:C group and on day 15 of H3 + Add and H3 + AP group. The promoted metabolism of H3 in mice induced by AP may be related to enhanced innate and adaptive response.

### AP-Adjuvanted H3 Vaccine Is Safe

**Fig. 9:**
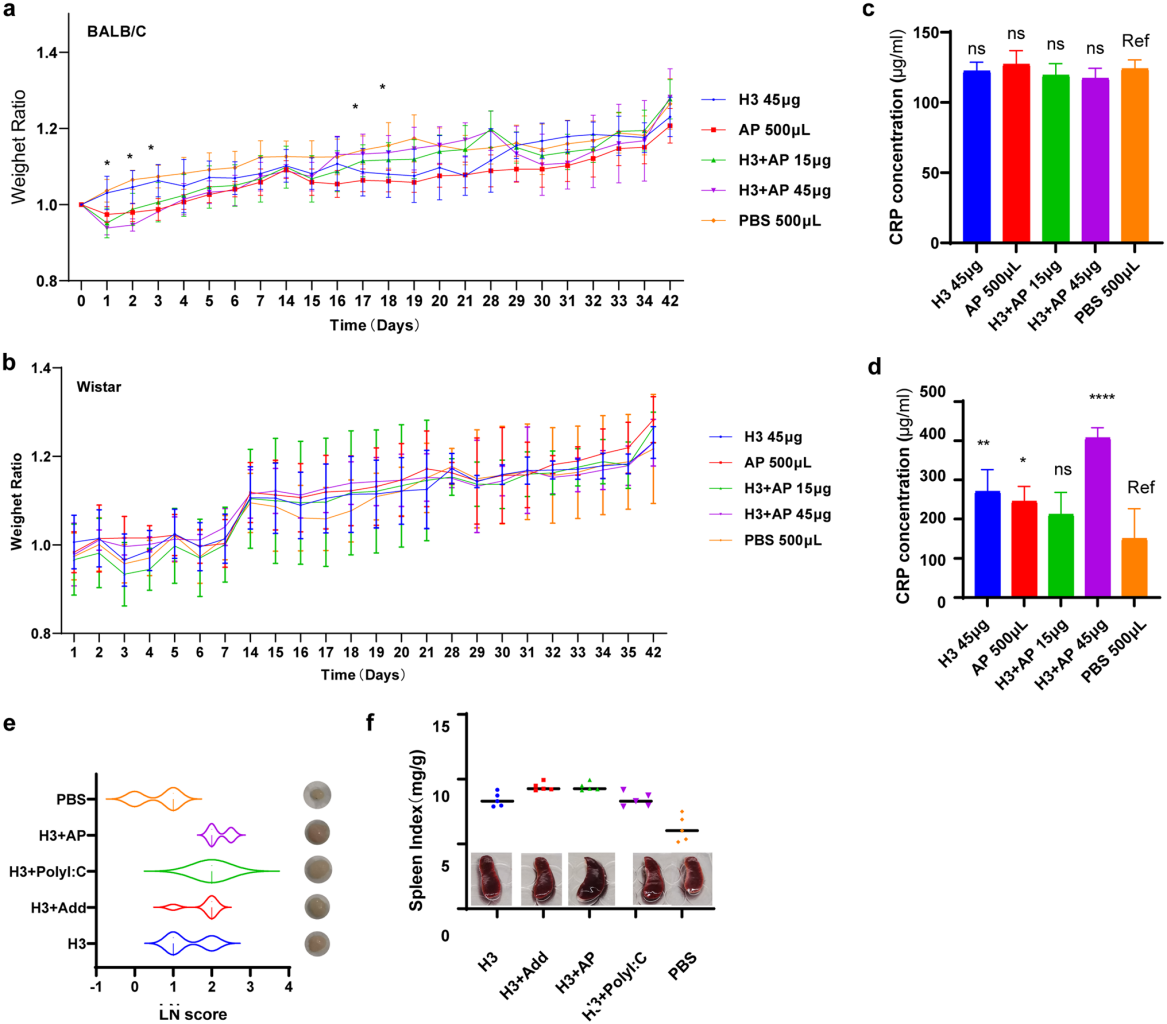
Evaluation safety of H3 + AP. Weight change of BALB/c mice **a** and Wistar rats **b**. BALB/c mice and Wistar rats were injected with 45 μg H3, 500 μL AP, 15 μg H3 + AP, 45 μg H3 + AP, and 500 μL PBS at day 0, 14, and 28. Animal weight was recorded everyday for 7 days and on the 14th day after each injection. Body weight ratio = daily mice weight/ weight on day 0. Serum CRP levels in BALB/c mice **c** and in Wistar rats **d**. LN score of BALB/c mice based on the hardness and size of LNs **e**. Spleen index of BALB/c mice **f**, spleen index was calculated as organ weight (mg) per gram **g** of mouse body weight. CRP was determined 7 days after the third injection and analyzed using Dunnett *t* test. *, significant; ns, not significant. All results are presented as means ± SD. Note: Ref represents the reference group

To further confirm that AP adjuvant has the potential to be used in influenza vaccines, we evaluated the safety of H3 + AP. After high-dose injection of H3, H3 + AP, AP, and PBS, BALB/c mice and Wistar rats in the experimental group (H3, H3 + AP, AP) lost weight on day 1 or 2 after each dose and then began to grow steadily until the next injection. The analysis of weight change in BALB/c mice showed a difference among groups mainly on the second day after each injection (Fig. 9a). There was no significant difference in body weight change of Wistar rats among these groups at each time point (Fig. 9b). Evaluation of CRP responses was carried out on day 7 after the last injection. The findings showed that serum CRP level of Wistar rats after administration of 45 μg H3 + AP (ninefold of the normal dose) was higher compared with that of the PBS group, and no significant difference was observed in the other groups (Fig. 9c, d), suggesting that H3 + AP has no significant effect on survival status of animal models.

BALB/c mice were administered with H3 combined with different adjuvants. The LN score of mice in the H3 group was higher compared with that in PBS group, and the LN score of mice in the adjuvant group (H3 + AP, H3 + Add, H3 + PolyI:C) was higher compared with that in the H3 group (Fig. 9e). The spleen index of mice in the adjuvant group and the H3 group were similar, but were both higher compared with that of PBS group (Fig. 9f).

## DISCUSSION

Only a few studies have explored use of w/o adjuvants and TLR agonists combined adjuvants in vaccines, especially the immune mechanism. In this study, we demonstrate that w/o adjuvant (AddaVax) and TLR agonist adjuvant (PolyI:C) combined adjuvant AP promotes antibody titer without toxic effects. Our data also demonstrate that AP induces a well-balanced and enhanced Th1 and Th2 immune response. The AP-enhanced adaptive immune response is induced by the release of local cytokines and chemokines at the injection site, local immune cell recruitment and the increased antigen uptake ability of DCs. These findings indicate that AP is a potential adjuvant.

Th2 and Th1 immune responses are believed to be related to protection; we found that H3 + AP could induce high levels of HI and IgG titers. A dose-dependent increase in H3 specific HI titer was observed in H3 and H3 + AP groups of BALB/c mice and Wistar rats. These findings are in agreement with previous influenza vaccine studies which have consistently shown dose-dependent vaccine effects (23, 24). Higher levels of IFN-γ, IL-4, IL-5, IL-6, IL-10, and IL-13 cytokine secretion from re-simulated splenocytes from H3 + AP immunized mice, these secreted cytokines have been associated with the most efficient induction of immune cells proliferation, differentiation, and activation to enhance both Th1 and Th2 responses (25). The enhanced Th1 and Th2 responses also confirmed by the activated number of IFN-γ- and IL-4-secreting immune cells induced by AP. In mice, IFN-γ secreted by Th1 cells and IL-4 secreted by Th2 cells regulates the isotypes of antibodies secreted. IFN-γ directs switching to IgG2a, whereas IL-4 directs switching to IgG1 (26); the higher IgG2a/IgG1 ratio induced by AP indicates a Th1-biased immune protective response. AddaVax enhances Th2-biased immune response similar to MF59 (27); PolyI:C induces Th1-biased response (28, 29). AP induces a well-balanced Th1 and Th2 immune response mainly through activity of AddaVax and PolyI:C component present in AP.

We found that high antigen-specific HI titer depends on H3 and AP being injected at the same injection site simultaneously; it is essential to explore the connection between local innate response and adaptive response. We have shown that AP induced a transient induction of cytokines at the injection site, indicating that the innate immune response at the injection site enhanced by AP promotes the body’s immune response to H3. AP induces expression of CCL2, CCL3, CCL5, CXCL1, and CXCL2 at the injection site, and these cytokines play a role in promoting recruitment of immune cells (30). It has been reported that the recruited immune cells further release pro-inflammatory cytokines, such as IL-12 and IFN-γ (31). In our study, the levels of inflammatory cytokines such as IL-6, IL-12, IL-17, IFN-γ and anti-inflammatory cytokines such as IL-4, IL-5, and TGF-β increased at the injection site within 5 days after H3 + AP injection. Inflammatory cytokines are critical for attracting neutrophils, monocytes, macrophages, and DCs to the injection site, so they are thought to play a key role in the innate immune response. TGF-β are produced to inhibit local inflammation by regulating pro-inflammatory mediators IL-6 responses (32). High level of TGF-β at the injection site stimulates migration of neutrophils, lymphocytes, and monocytes to induce chemotaxis (33). The immune microenvironment induced by AP facilitated immune cells migration and antigen transport from the injection site to the draining LNs, which is consistent with the immunofluorescence observed in LNs, and the increase in the number of T cells, DCs, and B cells in the LNs induced by AP. The first antibody response against antigens mainly occurs in LN draining the site of injection after acquisition of antigens by B cells (34–37).

The current study further explored the effect of AP on DCs and interaction between DCs and T cells. AP promoted the ability of immature DCs to take up antigen, mature, and release proinflammatory cytokines such as IL-12, which is essential for antigen presentation to the T cells. Activated mature DCs expressing T cell receptors and secreting cytokines effectively promote activation, maturation, differentiation of naive T cell, and induces immune response(38). AP enhanced cell surface expression of MHCII, CD40, CD80, CD86 on DCs, and it has been reported these surface markers mediate cross-reaction of antigen-specific CD4^+^ or CD8^+^ T cells, and effectively activate adaptive immune response (9, 39). AP facilitated a rapid immune response without antigen depot; thus, it can promote metabolism of antigens in the body (40).

Adjuvants may induce reactions at the injection site, such as redness, swelling, pain, and occasionally fatigue, malaise, and fever (41). Although inflammation was detected at the injection site of mice within 5 days after administration with AP, no redness and swelling were observed. The LN score, spleen index, CRP responses, and the weight change showed that AP was safe on experimental animals and showed a favorable benefit-risk ratio.

The current study provides a theoretical basis for use of AP as an adjuvant for influenza vaccine. The combined adjuvant AP which is a novel adjuvant platform presents with antigen delivery activity of AddaVax and immune enhancement function of PolyI:C. PolyI:C has potential use in therapeutic cancer vaccine (42), implying that AP can be used in a variety of vaccines. Although the findings of the current study are promising, research on signaling pathway mechanism is not conducted, and further studies should be conducted to determine that vaccines adjuvanted with AP are safe and can induce higher protective antibodies in the population.

## CONCLUSION

AP induces rapid and temporary response at the microenvironment of the vaccine injection site, and enhances antigen-specific cellular and antibody responses. Safety analysis showed that AP can be used as a potential adjuvant for vaccines.

## Abbreviations

APC: Antigen-presenting cell
BMDC: Bone marrow dendritic cells
BSA: Bovine serum albumin
CBA: Cytometric beads assay
CRP: C-reaction protein
CTL: Cytotoxic T lymphocytes
ELISA: Enzyme-linked immunosorbent assay
ELISpot: Enzyme-linked immunospot
FBS: Fetal bovine serum
GM-CSF: Granulocyte-macrophage colony-stimulating factor
GMT: Geometric mean titers
HA: Hemagglutinin
HAU: Hemagglutination unit
HRP: Horseradish peroxidase
i.m.: Intramuscular
LNs: Lymph nodes
MDCK: Madin-Darby canine kidney
OD: Optical density
PBS: Phosphate-buffered saline
PMA: Phorbol myristate acetate
RDE: Receptor-destroying enzyme
SRID: Single radial immunodiffusion
TGF-β: Transforming growth factor-beta
TLR: Toll-like receptor
TMB: 3,3′,5,5″-Tetramethylbenzidine
TPCK: *N*-Tosyl-l-phenylalanine chloromethyl ketone
w/o: Oil in water

## References

[1] Wiersma LC, Rimmelzwaan GF, de Vries RD (2015). Developing universal influenza vaccines: hitting the nail, not just on the head. Vaccines (Basel).

[2] Yeolekar LR, Guilfoyle K, Ganguly M, Tyagi P, Stittelaar KJ, van Amerongen G (2020). Immunogenicity and efficacy comparison of MDCK cell-based and egg-based live attenuated influenza vaccines of H5 and H7 subtypes in ferrets. Vaccine.

[3] Petrovsky N, Aguilar JC (2004). Vaccine adjuvants: current state and future trends. Immunol Cell Biol.

[4] Tregoning JS, Russell RF, Kinnear E (2018). Adjuvanted influenza vaccines. Hum Vaccin Immunother.

[5] Mbow ML, De Gregorio E, Valiante NM, Rappuoli R (2010). New adjuvants for human vaccines. Curr Opin Immunol.

[6] Kim EH, Woodruff MC, Grigoryan L, Maier B, Lee SH, Mandal P, et al. Squalene emulsion-based vaccine adjuvants stimulate CD8 T cell, but not antibody responses, through a RIPK3-dependent pathway, Elife. 2020;9. 10.7554/eLife.52687. 10.7554/eLife.52687 PMC7314549 32515732

[7] Choubini E, Habibi M, Khorshidi A, Ghasemi A, Asadi Karam MR, Bouzari S (2018). A novel multi-peptide subunit vaccine admixed with AddaVax adjuvant produces significant immunogenicity and protection against Proteus mirabilis urinary tract infection in mice model. Mol Immunol.

[8] Chen WH, Tao X, Agrawal A, Algaissi A, Peng BH, Pollet J, et al. Yeast-expressed SARS-CoV recombinant receptor-binding domain (RBD219-N1) formulated with alum induces protective immunity and reduces immune enhancement. bioRxiv. 2020. 10.1101/2020.05.15.098079. 10.1016/j.vaccine.2020.09.061 PMC7508514 33039209

[9] Hafner AM, Corthesy B, Merkle HP (2013). Particulate formulations for the delivery of poly(I:C) as vaccine adjuvant. Adv Drug Deliv Rev.

[10] Caskey M, Lefebvre F, Filali-Mouhim A, Cameron MJ, Goulet JP, Haddad EK (2011). Synthetic double-stranded RNA induces innate immune responses similar to a live viral vaccine in humans. J Exp Med.

[11] Matsumoto M, Funami K, Tanabe M, Oshiumi H, Shingai M, Seto Y (2003). Subcellular localization of Toll-like receptor 3 in human dendritic cells. J Immunol.

[12] Beljanski V, Chiang C, Kirchenbaum GA, Olagnier D, Bloom CE, Wong T (2015). Enhanced influenza virus-like particle vaccination with a structurally optimized RIG-I agonist as adjuvant. J Virol.

[13] Alqazlan N, Astill J, Taha-Abdelaziz K, Nagy E, Bridle B, Sharif S (2020). Probiotic lactobacilli enhance immunogenicity of an inactivated H9N2 influenza virus vaccine in chickens. Viral Immunol.

[14] Moriyama M, Chino S, Ichinohe T (2017). Consecutive inoculations of influenza virus vaccine and poly(I:C) protects mice against homologous and heterologous virus challenge. Vaccine.

[15] Moriyama M, Takeyama H, Hasegawa H, Ichinohe T (2017). Induction of lung CD8(+) T cell responses by consecutive inoculations of a poly(I:C) influenza vaccine. Vaccine.

[16] Shin JH, Noh JY, Kim KH, Park JK, Lee JH, Jeong SD (2017). Effect of zymosan and poly (I:C) adjuvants on responses to microneedle immunization coated with whole inactivated influenza vaccine. J Control Release.

[17] Hawksworth D (2018). Advancing Freund's and AddaVax adjuvant regimens using CpG Oligodeoxynucleotides. Monoclon Antib Immunodiagn Immunother.

[18] Jin B, Wang RY, Qiu Q, Sugauchi F, Grandinetti T, Alter HJ (2007). Induction of potent cellular immune response in mice by hepatitis C virus NS3 protein with double-stranded RNA. Immunology.

[19] Sadat SMA, Snider M, Garg R, Brownlie R, van DrunenLittel-van den Hurk S (2017). Local innate responses and protective immunity after intradermal immunization with bovine viral diarrhea virus E2 protein formulated with a combination adjuvant in cattle. Vaccine.

[20] China T.S.A.o., Laboratory animal—Guideline for ethical review of animal welfare. 2018. http://www.gb688.cn/bzgk/gb/newGbInfo?hcno=9BA619057D5C13103622A10FF4BA5D14. Accessed August 4th 2021.

[21] Lutz MB, Kukutsch N, Ogilvie AL, Rossner S, Koch F, Romani N (1999). An advanced culture method for generating large quantities of highly pure dendritic cells from mouse bone marrow. J Immunol Methods.

[22] Stewart VA, McGrath SM, Walsh DS, Davis S, Hess AS, Ware LA (2006). Pre-clinical evaluation of new adjuvant formulations to improve the immunogenicity of the malaria vaccine RTS, S/AS02A. Vaccine.

[23] Han HJ, Song MS, Park SJ, Byun HY, Robles NJC, Ha SH (2019). Efficacy of A/H1N1/2009 split inactivated influenza A vaccine (GC1115) in mice and ferrets. J Microbiol.

[24] Zingone F, Morisco F, Zanetti A, Romanò L, Portella G, Capone P (2011). Long-term antibody persistence and immune memory to hepatitis B virus in adult celiac patients vaccinated as adolescents. Vaccine.

[25] Vazquez MI, Catalan-Dibene J, Zlotnik A (2015). B cells responses and cytokine production are regulated by their immune microenvironment. Cytokine.

[26] Paydarnia N, Mansoori B, Esmaeili D, Kazemi T, Aghapour M, Hajiasgharzadeh K, et al. Helicobacter pylori recombinant CagA regulates Th1/Th2 balance in a BALB/c murine model. Adv Pharm Bull. 2020;10(2): 264–270. 10.34172/apb.2020.031. 10.34172/apb.2020.031 PMC7191242 32373495

[27] Han Q, Gao X, Chu Z, Wang X, EisaAddoma Adam F, Zhang S (2019). Truncated chicken MDA5 enhances the immune response to inactivated NDV vaccine. Vet Immunol Immunopathol.

[28] Sanchez MV, Elicabe RJ, Di Genaro MS, Germano MJ, Gea S, Garcia Bustos MF, et al. Total Leishmania antigens with Poly(I:C) induce Th1 protective response, Parasite Immunol. 2017;39(11). 10.1111/pim.12491. 10.1111/pim.12491 28901553

[29] Mehrizi AA, Rezvani N, Zakeri S, Gholami A, Babaeekhou L (2018). Poly(I:C) adjuvant strongly enhances parasite-inhibitory antibodies and Th1 response against Plasmodium falciparum merozoite surface protein-1 (42-kDa fragment) in BALB/c mice. Med Microbiol Immunol.

[30] Uyangaa E, Kim JH, Patil AM, Choi JY, Kim SB, Eo SK (2015). Distinct upstream role of type I IFN signaling in hematopoietic stem cell-derived and epithelial resident cells for concerted recruitment of Ly-6Chi monocytes and NK cells via CCL2-CCL3 cascade. PLoS Pathog.

[31] Gee K, Guzzo C, Che Mat NF, Ma W, Kumar A (2009). The IL-12 family of cytokines in infection, inflammation and autoimmune disorders. Inflamm Allergy Drug Targets.

[32] Boshtam M, Asgary S, Kouhpayeh S, Shariati L, Khanahmad H (2017). Aptamers against pro- and anti-inflammatory cytokines: a review. Inflammation.

[33] McCartney-Francis NL, Wahl SM (1994). Transforming growth factor beta: a matter of life and death. J Leukoc Biol.

[34] Wang HB, Weller PF (2008). Pivotal advance: eosinophils mediate early alum adjuvant-elicited B cell priming and IgM production. J Leukoc Biol.

[35] Kool M, Soullie T, van Nimwegen M, Willart MA, Muskens F, Jung S (2008). Alum adjuvant boosts adaptive immunity by inducing uric acid and activating inflammatory dendritic cells. J Exp Med.

[36] Lambrecht BN, Kool M, Willart MA, Hammad H (2009). Mechanism of action of clinically approved adjuvants. Curr Opin Immunol.

[37] Liang F, Lindgren G, Sandgren KJ, Thompson EA, Francica JR, Seubert A, et al. Vaccine priming is restricted to draining lymph nodes and controlled by adjuvant-mediated antigen uptake. Sci Transl Med. 2017;9(393). 10.1126/scitranslmed.aal2094. 10.1126/scitranslmed.aal2094 28592561

[38] Tai Y, Wang Q, Korner H, Zhang L, Wei W (2018). Molecular mechanisms of T cells activation by dendritic cells in autoimmune diseases. Front Pharmacol.

[39] Ott G, Barchfeld GL, Chernoff D, Radhakrishnan R, van Hoogevest P, Van Nest G (1995). MF59. Design and evaluation of a safe and potent adjuvant for human vaccines. Pharm Biotechnol.

[40] Pedersen GK, Worzner K, Andersen P, Christensen D (2020). Vaccine adjuvants differentially affect kinetics of antibody and germinal center responses. Front Immunol.

[41] Garcon N, Segal L, Tavares F, Van Mechelen M (2011). The safety evaluation of adjuvants during vaccine development: the AS04 experience. Vaccine.

[42] Ammi R, De Waele J, Willemen Y, Van Brussel I, Schrijvers DM, Lion E (2015). Poly(I:C) as cancer vaccine adjuvant: knocking on the door of medical breakthroughs. Pharmacol Ther.

